# Evolutionary Patterning: A Novel Approach to the Identification of Potential Drug Target Sites in *Plasmodium falciparum*

**DOI:** 10.1371/journal.pone.0003685

**Published:** 2008-11-10

**Authors:** Pierre M. Durand, Kubendran Naidoo, Theresa L. Coetzer

**Affiliations:** Department of Molecular Medicine and Haematology, University of the Witwatersrand and National Health Laboratory Service, Johannesburg, South Africa; University of Melbourne, Australia

## Abstract

Malaria continues to be the most lethal protozoan disease of humans. Drug development programs exhibit a high attrition rate and parasite resistance to chemotherapeutic drugs exacerbates the problem. Strategies that limit the development of resistance and minimize host side-effects are therefore of major importance. In this study, a novel approach, termed evolutionary patterning (EP), was used to identify suitable drug target sites that would minimize the emergence of parasite resistance. EP uses the ratio of non-synonymous to synonymous substitutions (ω) to assess the patterns of evolutionary change at individual codons in a gene and to identify codons under the most intense purifying selection (ω≤0.1). The extreme evolutionary pressure to maintain these residues implies that resistance mutations are highly unlikely to develop, which makes them attractive chemotherapeutic targets. Method validation included a demonstration that none of the residues providing pyrimethamine resistance in the *Plasmodium falciparum* dihydrofolate reductase enzyme were under extreme purifying selection. To illustrate the EP approach, the putative *P. falciparum* glycerol kinase (PfGK) was used as an example. The gene was cloned and the recombinant protein was active *in vitro,* verifying the database annotation. Parasite and human GK gene sequences were analyzed separately as part of protozoan and metazoan clades, respectively, and key differences in the evolutionary patterns of the two molecules were identified. Potential drug target sites containing residues under extreme evolutionary constraints were selected. Structural modeling was used to evaluate the functional importance and drug accessibility of these sites, which narrowed down the number of candidates. The strategy of evolutionary patterning and refinement with structural modeling addresses the problem of targeting sites to minimize the development of drug resistance. This represents a significant advance for drug discovery programs in malaria and other infectious diseases.

## Introduction

Malaria continues to be one of the most devastating human infectious diseases. *Plasmodium falciparum*, which causes the most virulent form of malaria, is responsible for 1 to 2 million deaths annually, mostly in children under the age of 5 years in predominantly resource-poor countries [1]. One of the major concerns in malaria research is the dire need for novel therapeutic strategies and the associated problem of parasite resistance. Generally, the drug discovery pipeline is one of attrition and less than one in every 50 potential projects proceed beyond the stage of clinical trials, which emphasizes the importance of appropriate drug target and lead compound selection [2]. Exacerbating this problem is the ever-present danger that resistance may develop, which in some cases may be rapid. The most dramatic example of this in the case of malaria was the emergence of resistance to pyrimethamine-sulfadoxine combination therapy in the 1960s, which occurred within 12 months of introducing the drug [3]. Pyrimethamine targets the dihydrofolate reductase (DHFR) enzyme in *P. falciparum* and DNA sequence analysis has identified five common point mutations in the gene that confer resistance [4]. Insights, therefore, that facilitate drug design and diminish the likelihood of drug resistance in the parasite would be invaluable.

A number of bioinformatic approaches may be used to identify essential amino acids in potential drug targets. The most commonly employed methods such as PSI-BLAST [5] and hidden Markov models [6], rely on protein sequence homologies and have been used to detect conserved local sequence motifs. Another approach that makes use of protein multiple sequence alignments (MSAs) is the evolutionary tracing (ET) method [7]. ET is based on the hypothesis that architecture-defining residues are mostly invariant, and traces these residues through a phylogenetic tree to guide investigators to structurally relevant sites. The protein homology-based methods are limited however, since some functional regions involve large contact areas that may only be apparent from 3D protein structures and are not obvious from primary sequence alignments [8]. Functional regions can also be organism-specific, particularly if the sequence homologies are low, and may not be clear from protein MSAs [9].

Homology methods do not include the evolutionary information available from nucleic acid sequences. Following the rapid progress in the field of molecular evolution and the vast amounts of genome sequence data available, it has been acknowledged that alternative approaches, such as those which make use of evolutionary analyses, should be applied at various points in the drug development pipeline [10]–[12]. This is particularly appropriate for pathogen drug design programs since whole genome data from several parasites (such as the *Plasmodium* and related apicomplexa genomes) are now available. To exploit this additional tier of information, pharmacophylogenomic analyses of genes and whole genome have been developed. Pharmacophylogenomics includes several evolutionary considerations that are important in drug target selection [10] such as: (i) orthologs versus paralogs, (ii) evolutionary dynamics of lineages and whole genes, and (iii) selection pressures that lead to rapid changes within genes. Differences in selective constraints between humans and animal models are also important for drug trials, where the drug target may be under different evolutionary dynamics in the animal model [10], [13].

In this study, we developed a novel approach, termed “evolutionary patterning” (EP), which makes use of the pattern of evolutionary change at individual codons across coding sequences to limit drug resistance by identifying the most constrained sites. EP can be combined with structural information and, like ET, is generic in nature. Nucleotide sequences contain information about the rate of evolution, which can be measured to determine the intensity of the selective force acting at a particular site in a protein. If a particular residue is critical to the structure or function of a protein, natural selection will remove any changes that occur at that site (purifying selection) at a rate that reflects its relative importance. These changes produce a pattern of evolution in extant taxa and can be used to identify residues under extreme purifying selection, which will potentially make the best drug target sites. Since evolutionary pressures act to maintain the most critical residues, it follows that mutations that confer drug resistance are unlikely to evolve at these sites.

*P. falciparum* glycerol kinase (PfGK) was selected as a model protein to evaluate the method. Glycerol kinase (GK) catalyzes the ATP-dependant phosphorylation of glycerol to glycerol-3-phosphate [14], a pivotal metabolite with multiple roles of providing the carbon backbone for glycerophospholipid synthesis, glycolysis or gluconeogenesis, and transporting reducing equivalents from the cytosol to the mitochondria for oxidative phosphorylation [14], [15].

A putative GK has been annotated in the *P. falciparum* genome database (PlasmoDB accession number PF13_0269). PfGK potentially phosphorylates glycerol, which enters the parasite from the host plasma via a *P. falciparum* aquaglyceroporin glycerol facilitator [16], [17]. Even though PfGK has not yet been validated as a drug target, this is not a prerequisite for testing our approach. PfGK fulfills many of the criteria that need to be considered when planning a drug discovery program [18]. Importantly it provides the essential backbone for phospholipid synthesis, which is required for membrane biogenesis during the extensive proliferation of asexual intraerythrocytic stage parasites [19]. Disruption of phospholipid synthesis impaired parasite development and cleared malaria infection in mice and monkeys [20], providing evidence for the essential role of GK in parasite metabolism. In addition, expression of the PfGK gene is upregulated in response to starvation and during gametocytogenesis when alternative carbon sources are required [21].

Other aspects that make PfGK an attractive potential drug target are that there are no known enzyme isoforms in the parasite and that human GK (HsGK) is not present in erythrocytes. Inhibitors such as 1-thioglycerol [22] and 5′-adenylyl imidodiphosphate [23] are available, which reflects the potential of the enzyme as a drug target. The crystal structure of an *Escherichia coli* ortholog (EcGK) has been resolved [24] and a putative 3D model of PfGK exists in the PlasmoDB database, which facilitates *in silico* structural analysis and drug design. In addition, in this study we provide the first evidence that PF13_0269 is indeed a GK ortholog, since recombinant PfGK was functionally active and phosphorylated glycerol in an *in vitro* assay.

The aim of this study was to investigate whether EP, together with structural data, can be used to (i) identify drug target sites that would limit the development of resistance and (ii) asses their suitability, using PfGK as an example. Based on EP data, six regions in PfGK were selected that contain residues under extreme purifying selection that are different to the human enzyme and thus represent potential drug target sites. Structural analysis revealed their location in the 3D PfGK model, which provided insight into the accessibility of drugs to these regions. It also elucidated the relative importance of these sites in the function of the enzyme. EP is a significant advance in the quest to identify potential therapeutic target sites and will serve as an additional filter to increase the likelihood of success of candidate proteins entering the drug development pipeline.

## Materials and Methods

### Sequence data and multiple sequence alignments (MSAs)

Twenty-eight orthologous GK protein and DNA coding sequences, for which there is laboratory or bioinformatic evidence, were retrieved from publicly accessible databases (Figure S1). True orthologs were identified from a previous study [25] or from pairwise alignments (data not shown). MAFFT [26] (algorithm G-INS-i, blosum62 matrix, gap-opening penalty of 1.53 and gap-extension penalty of 0.123) was used to perform the protein MSA and alignment accuracy was confirmed with the HoT test [27]. Nucleic acid MSAs were performed with DAMBE [28] using the protein sequence alignment as a template. BioEdit [29] was used to remove insertions from the alignments. MSAs may be found in Figures S2, S3, S4. In divergent nucleic acid sequences, saturation (>1 substitution at the same site) may lead to incorrect results and, where necessary, DAMBE was used to detect saturation.

### Phylogenetic analysis

**Figure 1:**
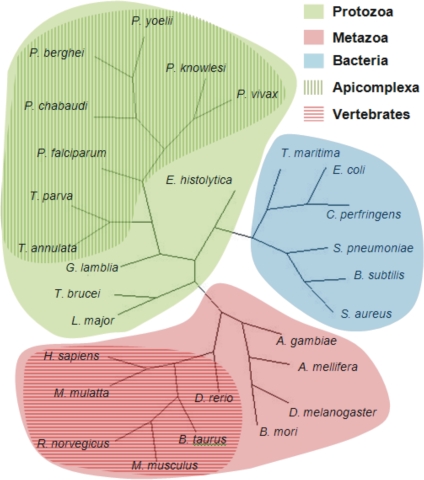
Phylogenetic reconstruction based on orthologous glycerol kinase protein sequences. The unrooted radial tree was generated using the maximum parsimony method. A maximum likelihood tree demonstrated the same topology. Bootstrapping provided >50% support for each node. The major branches comprising the protozoa, metazoa, apicomplexa and vertebrates were analyzed to identify differences in the patterns of sequence evolution between the four groups.

All phylogenetic analyses were performed with the PAUP* [30] software package. Phylogenetic analyses were performed on (a) a protein MSA of the 28 taxa, and (b) nucleic acid MSAs of major branches within the group of 28 taxa. Nucleic acid sequences were not used to construct a tree for the complete group of 28 taxa due to sequence saturation. In each case, maximum parsimony and maximum likelihood analyses were performed with 500 bootstrap replicates, and the consensus tree was saved. For the maximum likelihood phylogenetic tree construction the HKY85 evolutionary model was selected based on model testing with MODELTEST [31]. The branches within the group of 28 taxa that were analyzed separately were: (i) the protozoa (12 taxa) and a subgroup of protozoa, the Apicomplexa (8 taxa), and (ii) the metazoa (10 taxa) and a subgroup of metazoa, the vertebrates (6 taxa). The taxa that belong to each group or subgroup are shown in Figure 1. Phylogenetic analysis of these individual groups was necessary for further analysis with PAML and SLR (see below). TREEVIEW [32] was used to view the trees.

### Measurement of evolutionary change across GK coding sequences

The ratio (ω) of non-synonymous (dN) to synonymous (dS) nucleotide substitutions is used as a measure of evolutionary change and several methods exist to calculate these parameters, each with their own strengths and weaknesses [33]–[35]. Three categories of ω are typically considered: i) ω>1 reflects adaptive change or positive selection, ii) ω = 1 implies neutral selection, and iii) ω<1 indicates stabilizing or purifying selection, where nucleic acid substitutions that cause a change in the protein sequence (non-synonymous substitutions) reduce organism fitness and are removed by natural selection. Most genes exhibit a pattern of purifying selection (∼0.1<ω<1.0), however, the intensity of purifying selection varies between different genes [36], between individual codons within a gene [37] and across individual lineages within a tree [38]. Very small changes in ω (for example, a difference of 0.02) have biological relevance and, as expected, the greatest intensity of purifying selection occurs as ω approaches 0 [36]. Amino acid residues that are critical for protein structure and function are expected to be coded for by codons that are under the most intense purifying selection. To identify these codons, we introduced a fourth category in this study, which we called “extreme purifying selection”, and defined it as ω≤0.1. A value of 0.1 was chosen based on information from previous studies which compared the intensity of purifying selection between groups of genes [33], [36]. PAML (PAML and SLR are described below) was used to screen for codons with ω<1.0, estimated with a Bayes Empirical Bayes (BEB) probability of p>99%. To provide further evidence for the degree of purifying selection, codons identified with PAML were subjected to the site-wise likelihood ratio (SLR) test. Codons with ω≤0.1 and a statistical significance of p<0.01, adjusted for multiple comparisons, were placed in the “extreme purifying selection” category. Codons for methionine (ATG) and tryptophan (TGG) contain no potential synonymous substitutions and were excluded from the analysis.

### Evolutionary rate analysis with PAML and SLR

The PAML software package [39] (freely available at http://abacus.gene.ucl.ac.uk/software/paml.html) uses a phylogenetic analysis by maximum likelihood with several possible models to analyze evolutionary rates. Four models analyzed with the codeml algorithm were used: M0, M2, M7 and M8. M0 provided basic phylogenetic information such as branch lengths and transition/transversion ratios and allowed the consistency of these parameters to be monitored. M2 identified codons in the three ω categories ω>1.0, ω = 1.0 and ω<1.0. M7 identified the likelihood of the data fitting a β distribution with one ω value. M8 complimented M2, in that it was used to identify sites under positive selection (β distribution and ω>1.0). The probability of each codon fitting in a particular ω category (model M2) was determined by the BEB probability estimate. Likelihood ratio tests to compare models were unnecessary in this analysis.

The SLR method [40] combines the maximum likelihood phylogenetic approach developed by Nielsen and Yang [41] with the site-wise statistical test devised by Suzuki and Gojobori [42]. The SLR method determines ω for individual codons with a confidence interval for each ω value given by a p value, adjusted for multiple comparisons. The SLR software was kindly provided by Tim Massingham from the European Bioinformatics Institute, Hinxton Campus, United Kingdom.

PAML and SLR analysis were performed on the protozoa, apicomplexa, metazoa and vertebrate groups using the phylogenetic trees generated above. Protozoan and metazoan taxa were used to identify differences at individual codons in the GK coding sequence along these two lineages. PfGK and HsGK coding sequences were used as representatives of the protozoan and metazoan branches, respectively. The apicomplexa and vertebrate groups were used to observe the effects that changes to the number of taxa and clades may have had on the data.

### Identification of potential drug target sites in PfGK

By analyzing protozoan and metazoan clades separately, it was possible to identify differences in codons that were under extreme purifying selection in PfGK and HsGK. A pairwise alignment of the two protein sequences was performed with the Needleman and Wunsch algorithm [43].

### Method validation

The EP approach was validated in two ways:

1. The *P. falciparum* dihydrofolate reductase (*dhfr*) region of the dihydrofolate reductase-thymidylate synthase (*dhfr-ts*) fusion gene (accession number PFD0830w) was analyzed with PAML and SLR using the same Apicomplexa group (Figure 1) to determine the ω category of the codons in which point mutations conferring resistance to pyrimethamine occurred. *P. yoelii* was excluded from the analysis since *dhfr* and *ts* genes are not fused in this species and the evolutionary pressures may therefore be different. EP predicts that viable mutations are unlikely to occur at codons under extreme purifying selection and none of the five codons coding for the pyrimethamine-resistant mutations (C50R, N51I, C59R, S108N, and I164L) [3] were expected to have ω≤0.1 with a significant p value (<0.01, adjusted for multiple comparisons).
2. The crystal structure of EcGK revealed 19 functionally important amino acids [24] that are predicted to make contact with glycerol, ADP and Mg^2+^ ions. The 19 residues were located in the EcGK sequence of the MSA used in the phylogenetic analysis (Figure S2) and the corresponding PfGK residues identified. These sites were expected to fall into the extreme purifying selection category of ω due to their functional importance.

### PfGK structure

A putative PfGK 3D structure, based on the EcGK crystal structure, is available from the Plasmodium database version 5.4 (www.plasmodb.org/plasmo/home.jsp). Functionally important residues and potential PfGK target sites were identified and visualized using PyMOL v0.99 (www.pymol.org).

### Parasite culture

FCR-3 *P. falciparum* parasites were cultured *in vitro* using fresh human erythrocytes at 5% hematocrit and 10% AB plasma [44]. Cultures were incubated at 37°C in 93% N_2_, 5% CO_2_ and 2% O_2_ (Afrox, South Africa).

### Subcloning of the PfGK gene

*P. falciparum* DNA was extracted using phenol-chloroform and ethanol precipitation [45]. The full length 1506bp PfGK gene was amplified from 100ng DNA via PCR using 2.5U Expand High Fidelity *Taq* polymerase (Roche, Germany) and a forward primer: 5′-GATGGATCCATGAATGTCATATTAAGT-3′ containing a *Bam*HI (underlined) restriction site and a reverse primer 5′-GATCTCGAGTTATAACTGTATTAATGT-3′ containing a *Xho*I (underlined) restriction site. PCR was performed under the following conditions: 94°C, 2 min initial denaturation; 5 cycles: 94°C, 1 min; 35°C, 1 min; 72°C, 2 min; 30 cycles: 94°C, 1 min; 55°C, 1 min; 72°C, 2 min; and a final incubation at 72°C for 10 min. The amplified PfGK DNA sequence and pGEX-4T-2 (Amersham, UK) expression vector were digested with *Bam*HI and *Xho*I (Fermentas, Lithuania) and PfGK was ligated downstream of the Glutathione S-transferase (GST) gene sequence in the vector. Competent DH5α *E. coli* (Invitrogen, USA) were transformed with the plasmid construct and positive colonies were selected on Luria Bertani (LB) medium supplemented with 100 μg/ml ampicillin (Roche, Germany). The PfGK insert was verified using gene specific PCR, plasmid extraction, restriction enzyme analysis and DNA sequencing.

### Expression and purification of recombinant GST-PfGK (rPfGK)

rPfGK was expressed in Rosetta2 (DE3) *E. coli* (Novagen, USA) as a fusion protein with an N-terminal GST tag. Transformed cells were selected in LB medium containing 100 μg/ml ampicillin and 50 μg/ml chloramphenicol (Roche, Germany). 1/100 (v/v) culture was added to LB and cells were allowed to grow to an OD_600nm_≥0.6 at 37°C before induction with 1mM isopropyl thio-β-D-galactoside (IPTG, Promega, USA) for 6 hours at 37°C. rPfGK was predominantly expressed as insoluble protein trapped in inclusion bodies in *E. coli* and was purified with BugBuster® HT (Novagen, USA) as per manufacturer's instructions. rPfGK was denatured in 6M guanidine hydrochloride, 50mM Tris HCl pH 8.0, 100mM NaCl, 10mM EDTA and 10mM DTT (Merck, USA) [46] at 4°C overnight. The sample was diluted to 3M guanidine hydrochloride with refolding buffer (200mM Tris-HCl pH 8.0, 10mM EDTA, 1M L-arginine, 0.1mM PMSF, 2mM reduced glutathione, 0.2mM oxidized glutathione, Merck, USA). To refold the protein, it was dialyzed 8–10 times in mini dialysis tubes (Pierce, USA) against 10 volumes of the buffer for 5 min at room temperature. The refolded GST-PfGK was purified by affinity chromatography with GST°Mag™ Agarose beads (Promega, USA). Recombinant proteins were eluted from the beads with 100mM reduced glutathione, 50mM Tris-HCl, pH 8.0 elution buffer.

To improve the expression of soluble protein, 1/100 (v/v) of the antibiotic-selected cultures were added to either the Overnight Express™ Instant TB Medium (Novagen, USA) autoinduction system and allowed to grow to an OD_600nm_≥1.5 at room temperature or added to LB medium without antibiotics, grown to OD_600nm_≥1.0 at 37°C and induced with 0.4mM IPTG at room temperature to an OD_600nm_≥1.5. Cells expressing rPfGK were lysed with BugBuster® HT supplemented with the Protease Inhibitor Cocktail Set III (Novagen, USA). rPfGK was purified from the soluble *E. coli* protein fraction using GST○Mag™ Agarose beads as described above. Protein fractions were analysed by SDS-PAGE [47] and immunoblotting with a 1∶10000 (v/v) diluted anti-GST HRP-conjugated antibody (Amersham Biosciences, UK) and visualized with the SuperSignal® West Pico Chemiluminescent Substrate (Pierce, USA).

### PfGK Enzyme Activity

Purified rPfGK was quantitated using Coomassie Plus-the Better Bradford™ Assay kit (Pierce, USA). For the enzyme assay, EcGK (Worthington, UK) was used as a positive control and enzyme activity was measured in an ADP-coupled spectrophotometric assay as per manufacturer's instruction. Recombinant GST was used as a negative control and the blank had no rPfGK. Briefly, 0.5–1.0 μg purified rPfGK was added to a 3ml reaction mixture containing 0.7ml reagent solution (8.5mM disodium ATP, 1.22mM NADH, 2mM phosphoenolpyruvate, 28mM MgSO_4_, 26mM reduced glutathione, 5units pyruvate kinase, 10units lactate dehydrogenase, pH 7.4, Roche, Germany), 2.1ml carbonate-glycine buffer (0.4M glycine, pH 8.9, 45mM potassium carbonate, Roche, Germany) and 0.1ml 0.1M glycerol (Fluka, USA). Enzyme activity was detected as a decrease in NADH absorbance at 340nm over 20 min.

## Results

### Phylogenetic analysis

The maximum parsimony and maximum likelihood phylogenetic trees generated from the GK protein sequences of the 28 taxa demonstrated the same topologies (Figure 1). They were consistent with previous data [48] and the sequences were therefore suitable for further analysis. Nucleic acid sequence phylogenetic trees of the protozoan, metazoan, apicomplexa and vertebrate branches were used in the PAML and SLR analyses. Figures S5 and S6 contain the protozoan and metazoan phylograms, respectively. A logged output of the PAUP* data for generating phylogenetic trees for the protozoan branch is supplied as an example (Figure S7) and provides information regarding the g1 statistic, number of informative characters (parsimony analysis), treatment of gaps, heuristic search settings, distance matrices, tree statistics, tree lengths and bootstrap support.

### Evolutionary patterns for *P. falciparum* and human GK

**Figure 2:**
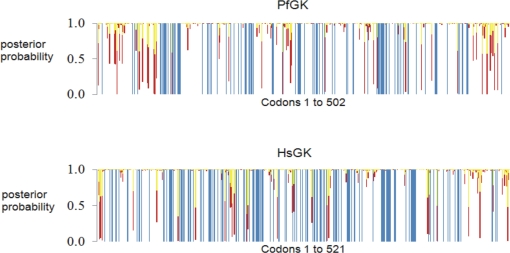
Posterior probabilities for four categories of ω across GK coding sequences. The Bayes Empirical Bayes posterior probability estimates (BEB) determined from the PAML analysis of the protozoan and metazoan clades were plotted for each category of ω across the *P. falciparum* (PfGK) and human (HsGK) GK coding sequences, respectively. Residues under extreme purifying selection (blue lines) are potential drug target sites. Categories for ω are indicated with colored bars: yellow (ω>1.0, positive selection), red (ω = 1.0, neutral selection), white (0.1<ω<1.0, purifying selection), and blue (ω≤0.1, extreme purifying selection). The ω<0.1 category was defined by combining PAML (BEB>99%) and SLR (p<0.01) data.

The PAML and SLR data were combined for the protozoan and metazoan clades to show a plot of the BEB posterior probability estimate for each category of ω across PfGK (protozoan) and HsGK (metazoan) coding sequences (Figure 2). For categories ω>1.0, ω = 1.0 and 0.1<ω<1.0 the PAML data were used. A summary of the PfGK data generated from the four models implemented with PAML is given in Table 1. The category ω≤0.1 was defined by the combined PAML and SLR data. PAML (Model M2) identified nearly 300 codons with ω<1.0 and a BEB probability estimate of >99%. Further analysis of these 300 codons with the SLR test identified 93 codons with ω≤0.1 and an adjusted p value <0.01 (SLR outputs can be found in Figures S8 (protozoa) and S9 (metazoa)). The 93 codons are represented in Figure 2 by the extreme purifying selection category with a BEB probability estimate of 100% (blue lines) and were used as a guide to select possible drug target sites. For both PfGK and HsGK, codons in the extreme purifying selection category spanned the entire coding sequence, although the 5̀ and 3̀ ends were significantly less conserved overall, and this was also more pronounced in PfGK. PAML and SLR analyses of the apicomplexa and vertebrate subgroups resulted in slightly more codons being placed in the extreme purifying selection category (112 in the apicomplexa and 121 in the vertebrate clades as opposed to 93 in the protozoan and 97 in the metazoan clades). Most of the additional codons were labeled as “constant” in the SLR analysis, indicating that the codons were identical across taxa.

**Table 1:** Parameter estimates, log-likelihood values and selective pressures across codons for *P. falciparum* glycerol kinase under models of variable ω ratios.

Model	ω categories	ℓ	κ	Parameters	Neutral/positively selected sites
M0	1	−4568.9	1.8	ω = 0.028	None
M2	3	−4543.5	2.0	ω = 0.02 (p = 0.95), ω = 1.0 (p = 0.04), ω>1.0 (p = 0.00)	N2 V16 E22 I25 S28 T52 N56 I70 K71 K76 I92 P149 E226 L236 N237 E277 S331 E373 V407 D461 K492
M7	β distribution	−4520.8	1.9	*p* = 0.30708, *q* = 7.68249	Not allowed
M8	β distribution, ω>1.0	−4520.8	1.9	*p* = 0.30708, *q* = 7.68255 and ω>1.0 (p = 0.00)	None

**Figure 3:**
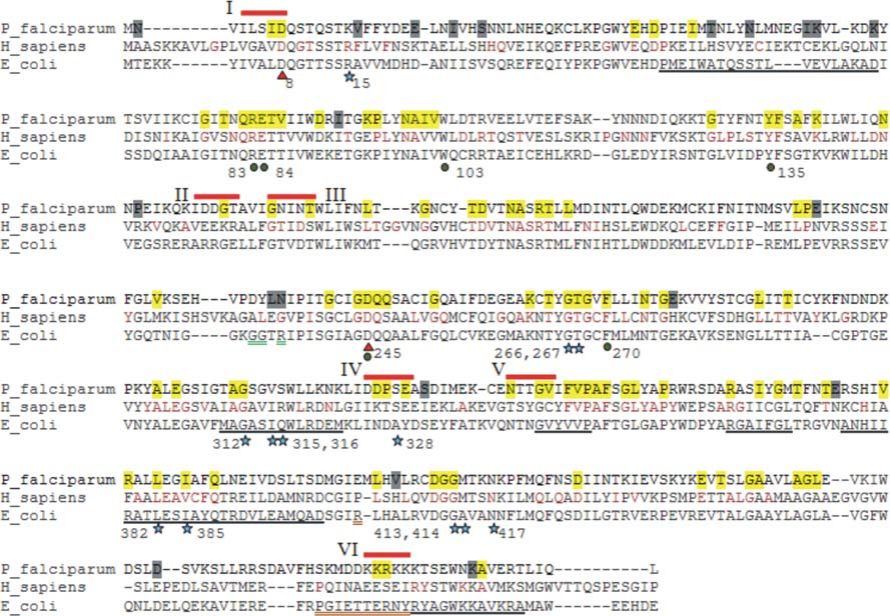
Multiple sequence alignment of PfGK, HsGK and EcGK. To identify differences in selective constraints, PfGK and HsGK were analyzed separately, as part of protozoan and metazoan clades, respectively, and compared. Residues under extreme purifying selection are highlighted in yellow in PfGK and are in brown font in HsGK. Residues highlighted in gray in PfGK are under neutral or positive selection. Sites that bind glycerol (green circle), ADP (blue star) and magnesium (red triangle) were determined from the crystal structure of EcGK [24]. Corresponding residues in PfGK were determined form the MSA. Residues are numbered according to the PfGK sequence. Potential drug target sites are indicated (red bar) and labeled with Roman numerals. Underlined regions in EcGK are implicated as follows: intersubunit interactions (black), FBP regulatory site (green) and IIA^Glc^ regulatory site (orange).

Models were implemented with the codeml algorithm in the PAML package. Under the M2 model, there were >300 codons with ω<1.0 and a posterior probability of >99% (not listed in table). These were subjected to the SLR test for further categorization of the intensity of purifying selection (see text). There were 21 neutral or positively selected sites (ω≥1) with a Bayes Empirical Bayes (BEB) posterior probability of >50%, although none of these were statistically significant at 95%. Under the M8 model, no statistically significant positively selected sites were identified. For M7 and M8, *p* and *q* are the shape parameters of the β distribution. ℓ = log-likelihood estimate, κ = transition/transversion ratio, p = BEB posterior probability. Numbering corresponds to the sequence alignment in Figure 3.

PAML (Model M2) also identified 21 sites (Figure 2 and Table 1) under neutral (red lines) or positive (yellow lines) selection (ω≥1) although these were not statistically significant at the 99% level. The reason for this may be that the natural selective forces are weak or there may be too little evolutionary information present in the available sequences to make the findings significant. The lack of conservation at neutral or positively selected codons indicates that these sites are unlikely to be critical for GK function. Drugs targeted against these residues may therefore fail to disrupt enzyme activity, and even if they did, mutations conferring resistance are likely to evolve.

Some residues in PfGK under extreme purifying selection were not part of any recognized functional domains (Figure 3). However, these may be important in protein folding or play a role in the stability of the 3D molecular structure. In addition, some residues in this category were not classified as such in HsGK, and vice versa. The reason for this is most likely the differing selection pressures acting on the two proteins.

### Validation of the EP approach

1. The SLR logged output following analysis of the edited MSA of orthologous *dhfr* coding sequences is available in Figure S10. The ω category for *P. falciparum* codon 103 corresponding to the key mutation S108N that confers drug resistance was that of weak purifying selection with an adjusted p value that was not statistically significant. The remaining four codons implicated in drug resistance either had no synonymous substitutions (codon 45) or were not significantly different from a random alignment (codons 46, 54, and 159), implying that they are either under weak purifying, neutral or positive selection. As predicted by EP, none of the five codons that mutated to provide resistance against pyrimethamine in PfDHFR were under extreme purifying selection.
2. Twelve of the 19 functionally important residues that are invariant in GK and which had been included in the MSA (Figure S2) were categorized as being under extreme purifying selection (Figure 3). This was taken as further validation of the EP approach. The remaining seven functional residues did not fall into the extreme purifying selection category for the following reasons: (i) W103 (glycerol binding) and M414 (ADP-binding) were excluded since there is only one possible codon for each, and (ii) residues K15, V315, S316, S328 and N417 (all ADP-binding) are variable in a MSA [49], indicating that non-synonymous substitutions are present in the coding sequences, which are therefore, highly unlikely to be under extreme purifying selection. The variability in these four ADP-binding sites may relate to the fact that the ADP-binding pocket is formed by 12 amino acids and that not all of these are critical.

### Selection of potential drug target sites

For the purposes of limiting resistance and providing an adequate target site, regions containing several amino acids under extreme purifying selection would be ideal. Examples of such regions in PfGK are residues 82–86 (QRETV), 99–102 (NAIV), 244–247 (GDQQ), 266–270 (GTGVF) and 345–349 (FVPAF) (Figure 3). However, most of these amino acids are identical in HsGK and these sites were therefore not selected. Other regions were chosen using the following criteria: (i) they contained one or more residues with ω≤0.1, (ii) the remaining residues had low ω values (<0.2), and (iii) PfGK and HsGK shared no more than two identical residues. The exact length of the region would depend on its potential druggability, an analysis of which is beyond the scope of this paper, but for demonstration purposes an arbitrary length of five residues was used. For example, in region I in PfGK, I7 and D8 are under extreme purifying selection. D8 binds Mg^2+^ and is the same in HsGK, but I7 aligns with V in the human sequence. Residues flanking the amino acids under extreme purifying selection were examined and the region was extended by three residues (ω values ≤0.14) towards the N-terminus. Six regions (labeled I to VI) were selected that may be useful as potential drug target sites (Figure 3, Table 2).

**Table 2:** Summary of the location and function of potential PfGK drug target sites.

Region	Color^1^	Sequence	Functional domain^2^	Location	Drug Target
I	Magenta	ILSID	Mg^2+^-binding	Catalytic Cleft	No
II	Red	IDDGT	None	Surface	No
III	Black^3^	GNINT	ATPase core	Core	No
IV	Blue	DDPSE	ADP binding	Catalytic Cleft	Yes
V	Orange	NTTGV	Subunit interaction	Surface	Yes
VI	Green	KKRKK	Allosteric regulation	Surface	Yes

**Figure 4:**
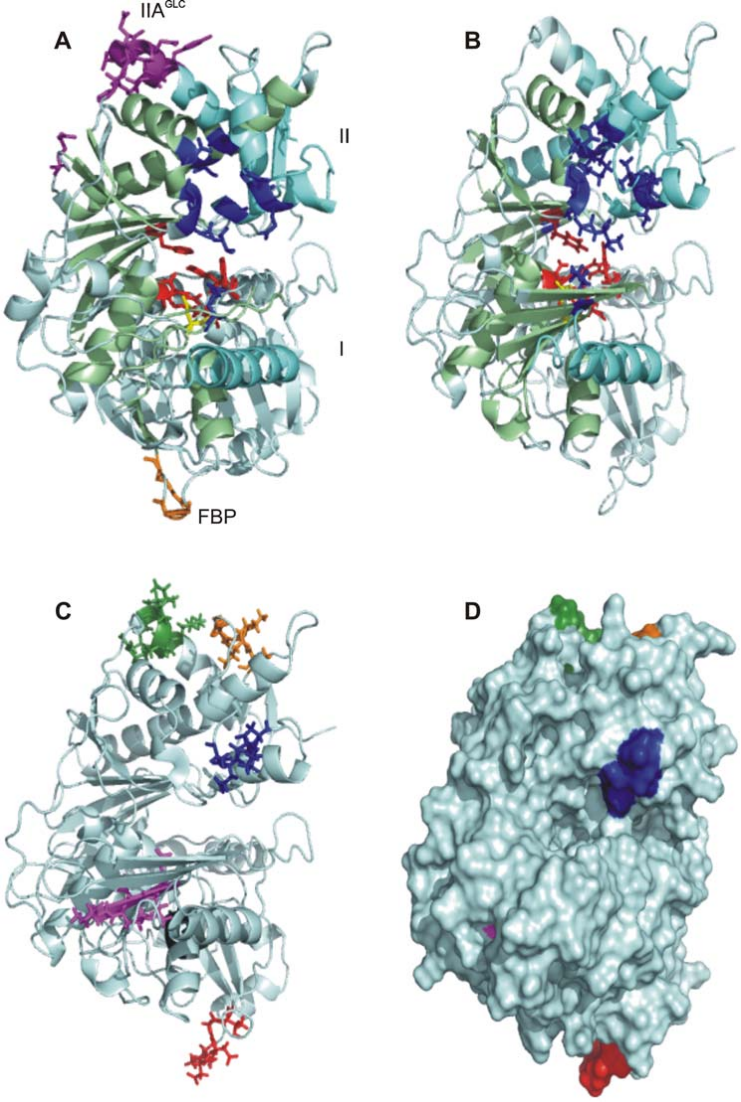
EcGK and PfGK models. Models A (EcGK) and B (PfGK) are displayed as ribbon structures with functional residues illustrated as sticks. (A) An EcGK monomer with residues involved in binding to ADP (blue), Mg^2+^ (yellow), glycerol (red), FBP (orange) and IIA^GLC^ (purple) [24], [70]. Positions of Domains I and II are labeled. α Helices and ß sheets within Domains I and II form the ATPase domain, shaded in light green and areas involved in subunit interactions are shaded in aquamarine. (B) The PfGK model showing functional residues based on EcGK. (C) PfGK ribbon structure and (D) surface model showing potential drug target sites. Residues within each region are summarized in Table 2 and are highlighted as follows: Region I (magenta), Region II (red), Region III (black), Region IV (blue), Region V (orange) and Region VI (green). Region III is below Region I and is difficult to visualize in the ribbon configuration (C) since it is partly obscured by one of the helices. It is within the core of the molecule and cannot be seen on the surface PfGK model (D).

1 Colors of each region correspond to those in Figures 4C and 4D.

2 GK functional and regulatory domains are illustrated in Figures 4A (EcGK) and 4B (PfGK).

3 This region is in the core of the molecule and cannot be seen on the surface model (Figure 4D).

### Structural analysis

GK is a member of the ATPase superfamily that shares a common ßßßαßαßα tertiary fold domain [50]. Under physiological conditions, EcGK exists as functional dimers and tetramers in equilibrium, as well as inactive tetramers, each composed of identical monomers [51]. Each monomer (Figure 4A) consists of two major domains separated by a deep active site cleft and contains six subdomains involved in the ATPase core (light green), intersubunit interactions (aquamarine) and substrate binding (red). EcGK is independently and non-competitively inhibited by two allosteric effectors: the glycolytic intermediate fructose 1,6-bisphosphate (FBP) and IIA^GLC^, a component of the *E. coli* phosphoenolpyruvate:glycose phosphotransferase system [24].

A putative PfGK structural model indicating functionally important residues based on homology to EcGK is shown in Figure 4B. To evaluate the functional significance of the six potential drug target sites, the regions were mapped to the PfGK structure (Figures 4C and 4D). All the regions, except for Region II, were found in functionally important domains as summarized in Table 2.

Region I (magenta) represents a β sheet at the N-terminus of PfGK and is located within the catalytic cleft. It shares a common Mg^2+^ binding site (D8) with HsGK. Region II (red) has two negatively charged residues and represents a loop structure between an α helix and β sheet at the surface of the molecule. It does not represent any known functional or structural GK domain. Region III (black) forms an α helix-turn-β sheet structure and is located within the hydrophobic core of the molecule, below the glycerol-binding domain. It represents part of the ATPase core in Domain I. Region IV (blue) is part of a loop between two α helices and is located at the entrance to the catalytic cleft. S328 participates in ADP-binding and the adjacent P327 may confer conformational stability to the ADP-binding site. Region V (orange) represents a loop structure on the surface of the molecule and contains residues involved in intersubunit interactions in EcGK dimerization. Region VI (green) is situated on an α helix and is the site for EcGK IIA^GLC^-allosteric regulation. In PfGK, Region VI forms part of a low complexity region which loops out on the surface of the molecule. All five residues within Region VI are positively charged and share no similarity with the corresponding region of HsGK, which has three negatively charged residues.

### Recombinant PfGK is active

**Figure 5:**
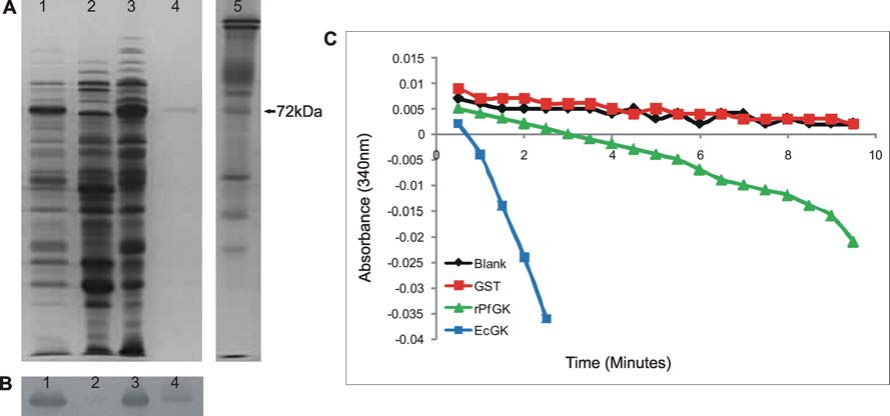
rPfGK purification and enzyme activity. (A) SDS PAGE analysis and (B) immunoblot analysis showing the expression and affinity purification of rPfGK from the soluble protein fraction of *E. coli* cells. Lane 1: total *E. coli* protein, Lane 2: soluble protein fraction, Lane 3: insoluble protein fraction, Lane 4: purified rPfGK, Lane 5: red cell membrane marker. (C) The oxidation of NADH to NAD^+^ in the glycerol kinase coupled enzyme reaction measured at 340nm shows active rPfGK (green) compared to the blank (black), negative control GST (red) and the positive control EcGK (blue).

The expressed rPfGK protein was predominantly insoluble and trapped as inclusion bodies in *E. coli* (Lane 3, Figure 5A). Refolding and purification of rPfGK from the inclusion bodies yielded inactive enzyme (data not shown). Expression of soluble rPfGK was improved to produce ∼100ng purified rPfGK from a 100ml culture (lane 4, Figure 5A). The rPfGK protein migrated at ∼72kDa on a polyacrylamide gel and immunoblot analysis (Figure 5B) confirmed the presence of the GST tag on rPfGK. Enzyme assays from three independent purifications showed that rPfGK was active and phosphorylated glycerol (Figure 5C).

## Discussion

Evolutionary-based approaches for the identification of structurally and functionally important residues in protein sequences have focused on sequence homology, phylogenetic tracing of structurally-important residues (ET) and confirmation of orthology (as opposed to paralogy). In addition, an analysis of evolutionary rates has been suggested as a means to identify genes that are subject to adaptive evolution and should therefore be avoided as drug targets [10]. In this study a new approach, termed evolutionary patterning (EP) was introduced with the aim of identifying and assessing the suitability of potential drug target sites from the point of view of limiting the evolution of mutations conferring drug resistance. Furthermore, using PfGK as an example, structural modeling was used to refine the selection of potential target sites by assessing their functional and structural significance. The putative PfGK was cloned and the recombinant protein was active, verifying the database annotation and justifying it as a potential target.

Validation for the EP approach was provided by PfDHFR data. EP theory predicted that none of the codons that evolved mutations providing resistance against pyrimethamine would be under extreme purifying selection, and this was demonstrated in this study. It is also interesting to note that none of the drug-binding residues in the PfDHFR enzyme (I14, C15, D54, F58, P113 and I164) [52] are under extreme purifying selection (data not shown), and these residues would not have been selected as drug target sites using the EP approach. As evident from the PfDHFR example, future drug development programs that target enzyme active sites without taking into account the evolutionary dynamics run the risk of failure.

### Potential drug target sites identified by EP and structural mapping

Analysis of the evolutionary patterns of PfGK residues in comparison to the human enzyme led to the selection of six regions that included sites that are not prone to viable mutation. Differences between HsGK and PfGK sequences were favored when selecting potential drug target sites since this would, theoretically, diminish drug side-effects. These sites were further evaluated by structural modeling of PfGK. Of the six regions identified, region IV (blue in Figures 4C and 4D) appears the most favorable. It has a number of features that make it an attractive drug target: (i) one residue (S328) has a predicted function (ADP-binding) as determined from MSAs and the crystal structure of EcGK [24], (ii) the target site is located at the entrance to the catalytic pocket, (iii) D326, P327 and E329 are adjacent to the S328 ADP-binding residue and are under extreme purifying selection in the parasite, indicating that resistance mutations in this region are unlikely to develop, and (iv) three out of the five residues in PfGK are different to the human enzyme, which, favors selective targeting of the parasite.

Analysis of the other potential drug target sites revealed that regions I, II and III may not be good candidates. Region I (magenta in Figures 4C and 4D) binds Mg^2+^ within the catalytic pocket. Its location makes it an attractive target site, however, small Mg^2+^ ions can easily enter the catalytic pocket whereas a larger potential lead compound may not be able to gain access. Region II (red in Figure 4C and 4D) is on the surface of the molecule making it accessible to a drug, however, it is relatively isolated from the catalytic domain and is not involved in any functional interactions. Targeting this site may therefore not inhibit the enzyme, although the possibility of long range conformational changes induced by the binding of a drug cannot be ruled out. Region III (black in Figure 4C) is within the hydrophobic core of the molecule and may be inaccessible to drugs.

Region V (orange in Figures 4C and 4D) and VI (green in Figures 4C and 4D) are exposed on the surface of the molecule and in EcGK, they participate in intersubunit and allosteric interactions, respectively. The oligomeric state of the active PfGK enzyme, as well as potential regulation by allosteric effectors, is not known. However, regions V and VI are in close proximity to each other and could collectively form one drug target site.

The structural modeling of PfGK illustrates how it complements and refines the EP approach by eliminating sites that are not useful. However, it should be noted that this type of analysis represents an initial step, since the druggability of each site requires evaluation by medicinal chemists [53], [54].

### EP: strengths and advantages

The fundamental strength of EP is that the intensity of purifying selection on individual codons is measured and the most evolutionary constrained amino acids are identified. Residues that are under extreme purifying selection imply that mutations at these sites dramatically decrease organism fitness and would be removed by natural selection. Viable mutations leading to drug resistance are therefore less likely to evolve, which will effectively increase the lifespan of therapeutic agents targeted to these sites. In addition, sites under positive or neutral selection are likely to adapt to drug pressures and should be avoided as targets.

Quantifying the pattern and intensity of selection across codons within a gene has additional advantages. For example, it is important to know the ω values of residues adjacent to amino acids under extreme purifying selection if a particular region is considered a potential drug target site. Furthermore, lead compounds are unlikely to interact exclusively with a stretch of contiguous amino acids. It is more likely that, due to protein folding and subsequent 3D structure, a drug makes contact with several non-contiguous residues. It would be important to identify the selection pressure at these residues since amino acid changes at these sites may dramatically affect drug binding. Information gathered with EP is therefore valuable in the iterative process of drug design: (i) appropriate targets sites are identified and selected, (ii) lead compounds are designed to interact with the selected sites, (iii) *in silico* docking identifies all the potential contact sites, (iv) contact sites are evaluated with EP, (v) lead compounds are modified if necessary such that contacts are not made with sites that are prone to change, and (vi) the process may be repeated until the drug-protein interactions are optimized or the potential target site is abandoned.

The PAML and SLR methods can detect very small changes in ω at individual codons and in different lineages, which can be statistically and biologically significant [36]. In this respect, EP can be applied to subgroups delineated by branch points in a phylogenetic tree to detect such differences. This is important for the design of antiprotozoal drugs where differences between host and parasite orthologs are exploited to target the parasite selectively and minimize side-effects [55].

Genome-wide analyses of *P. falciparum* diversity has identified vast numbers of SNPs (single nucleotide polymorphisms) located throughout the genome [56], [57]. This has raised concerns regarding drug development since drugs targeted to polymorphic sites could rapidly lead to the emergence of resistant strains. However, the EP approach would overcome this problem since polymorphic sites that lead to changes in amino acid residues will not have ω≤0.1, and will therefore not be selected as potential drug target sites.

Finally, to maximize the robustness for predicting target sites, EP combines two statistical estimates of ω. First, the maximum likelihood codon substitution model developed by Yang and Nielsen [58], which takes into account the transition/transversion ratio and nucleotide usage bias, is used to determine the ω category (for example, ω<1.0). The BEB probability estimate is used as a measure of the accuracy of that inference and only codons with ω<1.0 and a probability estimate with a confidence interval greater than 99% are selected (model M2 in the codeml algorithm). Second, the sitewise test developed by Suzuki and Gojobori [42] is used to perform a likelihood ratio test to identify codons with ω values significantly different from 1.0 with an adjusted p<0.01 [40]. Codons with ω≤0.1 and which satisfy both statistical criteria are investigated as potential drug target sites.

### EP: limitations and potential pitfalls

EP is limited by the availability of adequate sequence data. A discussion of the selection of an optimal data set is beyond the scope of this paper and the reader is referred to Yang [59] for an authoritative treatment of the topic. However, one aspect that emerged from this study is that care must be taken when selecting phylogenetic subgroups for the purpose of comparing host and parasite proteins. Although PAML and SLR are designed to account for phylogenetic parameters like branch lengths, we investigated whether differences in the evolutionary relationships between taxa in protozoan and metazoan clades might have influenced the results. To do this, we used a subset of the taxa in each of the two groups (apicomplexa for the protozoan clade and vertebrates for the metazoan clade) and repeated the analyses, which resulted in slightly more codons being placed in the extreme purifying selection category. Most of the additional codons were labeled as “constant” in the SLR analysis, indicating that the codons were identical across taxa. This suggests that the reason for the identification of more codons in the apicomplexa and vertebrate clades was the result of using fewer taxa and smaller evolutionary distances in the phylogenetic tree. The fact that there were only minor differences is testament to the power of PAML and SLR. Nevertheless, this does indicate that optimal input data must be used, particularly in studies such as this one where small differences in the evolutionary constraints of a protein are being investigated.

Another possible limitation of EP is that some potential drug target sites may be missed due to the rigorous statistical criteria imposed by the method. However, when one considers the time span of drug development pipelines, it seems more appropriate to run the risk of missing potential sites rather than permitting the inclusion of residues that are likely to mutate.

One issue that the current description of EP does not account for is the possibility that drug resistance may develop in cases where mutations at the drug target site are combined with compensatory mutations elsewhere in the protein. However, this problem can be addressed by investigating the potential co-evolution between codons. Methods to measure correlated evolutionary rates within a protein exist and may be incorporated into an EP analysis [60]. In addition, EP cannot account for heterotachy, a phenomenon that describes shifts in evolutionary rates over time [61]. However, these changes occur over extended evolutionary time periods and may therefore have a limited impact on drug viability.

A potential pitfall of EP is that database sequences will typically be used. Databases occasionally contain erroneous information [62] and this possibility should be considered when results are not in keeping with biological expectations. In the process of performing this study, a number of annotation errors were detected, which would have led to incorrect results. For example, to investigate the consequences of inadvertently using a paralog, the chicken GK2 (NCBI accession number XP_416788.2) was included in the analysis of the metazoan clade (data not shown). This greatly diminished the number of codons under extreme purifying selection (from 121 to 6) emphasizing that only true orthologs must be used, which is in keeping with previous reports [10], [13].

For the purposes of drug design, the temptation to screen for potential target genes by applying evolutionary analyses to whole coding sequences represents a serious potential pitfall. Estimating ω for a whole gene will provide misleading information since ω may vary considerably at individual codons. A recent demonstration of this is the *MHC* (major histocompatibility) gene, which demonstrates an average ω of weak purifying selection (0.6) although the selective constraints on codons vary from positive selection (ω = 4.7) to extreme purifying selection (ω≤0.1) [63].

### EP: applications

EP is generic in nature and can potentially be applied to any set of coding sequences, provided there are sufficient data to make the results statistically meaningful. For drug development against the malaria parasite, there is the additional advantage that approximately ∼60% of the predicted proteins in *P. falciparum* have little or no homology to known proteins [64]. These proteins are annotated as “hypothetical” in PlasmoDB and have orthologs in other *Plasmodium* species, which may be used for comparison. Applying EP in this context will identify residues under extreme purifying selection in proteins with no human ortholog, which will make them attractive drug targets.

EP analysis of PfGK demonstrated that many of the sites under extreme purifying selection have functional or structural importance. This finding suggests that EP may also be used to identify these residues in uncharacterized “hypothetical” proteins in *P. falciparum*, or indeed, any other organism. This potential application is currently being investigated in our laboratory and represents a rapid, cost-effective way of advancing our knowledge of these proteins. In the absence of EP, the identification of structurally and functionally important residues depends on crystal structures, analysis of protein-protein interactions and site-directed mutagenesis.

It is envisaged that EP will be used as an initial step to identify the most suitable drug target sites in a protein. This information can subsequently be utilized by medicinal chemists further along the drug development pipeline to initiate the design of appropriate compounds.

### Software available for EP analysis

A number of methods exist for estimating ω [58], [65], [66] and although PAML and SLR were used in this study, there are many other excellent and equally suitable software packages. A comprehensive list of the available software for analyses in molecular evolution is maintained by the Department of Genome Sciences, University of Washington, Seattle, USA and may be found at http://evolution.genetics.washington.edu/phylip/software.html. For most applications the maximum likelihood framework appears to be as good or superior to other methods, however, the accuracy of the estimation depends on a number of factors such as the true ω value and the transition/transversion ratio [58], and alternative methods should be considered when these parameters are unexpectedly high.

The functional divergence at amino acid sites among phylogenetic clusters can also be identified and mapped to tertiary structures with software like DIVERGE (Detecting Variability in Evolutionary Rates among Genes) [67]. This enables the user to identify sites that have different evolutionary, and therefore, functional constraints in two branches within a tree (for example, host and pathogen branches), thereby focusing on regions that are specific to the pathogen cluster. Similarly, “tertiary windowing” allows the user to select structural regions of interest and asses the evolutionary constraints at the chosen sites, which has the advantage of examining the 3D structure of a protein in order to select drug target sites prior to performing a comprehensive evolutionary analysis [68]. DIVERGE, tertiary windowing and related software will be particularly useful for selecting appropriate sites by avoiding residues that are likely to change, however, the ability to detect amino acid sites that are under the most extreme purifying selection is limited.

A noteworthy point applicable to almost all methods is that current evolutionary analysis makes the assumption that synonymous substitutions are neutral and therefore have no bearing on the interpretation of ω. Although this is not necessarily true [69], the selective forces acting on synonymous changes are poorly understood. Until such time that evolutionary models are developed to account for this, the assumption of neutrality at synonymous sites remains.

### Conclusions

EP is a novel approach that makes use of the evolutionary dynamics of genes and proteins for the purposes of selecting suitable drug target sites. EP identifies amino acids that are under the most extreme evolutionary constraints. These sites are predicted to be the most appropriate drug targets since they are structurally and/or functionally essential, and are the least likely to undergo viable mutations, thereby limiting resistance and increasing the lifespan of a drug. Although EP is applicable on its own, molecular modeling compliments and refines the process of target site selection by assessing the topography and functional significance of the potential sites. EP, combined with structural analysis, addresses a major obstacle to efficient drug design: that of appropriate target site selection to limit the evolution of resistance. This approach may be used as an inexpensive first-line strategy for supplying medicinal chemists with potential drug target sites, which could significantly reduce the attrition rate and lead to more effective therapeutic agents against malaria and other infectious diseases.

## Supporting Information

Figure S1 Sequences were retrieved from three databases: NCBI (http://www.ncbi.nlm.nih.gov), OrthoMCL (http://www.orthomcl.org/cgi-bin/OrthoMclWeb.cgi), and PlasmoDB version 5.3 (http://plasmodb.org).
(0.05 MB DOC)

Figure S2 The multiple sequence alignment includes all 28 taxa used in this study and was performed with MAFFT.
(0.02 MB TXT)

Figure S3 The multiple sequence alignment was performed with DAMBE using the protein multiple sequence alignment as a template.
(0.02 MB TXT)

Figure S4 The multiple sequence alignment was performed with DAMBE using the protein multiple sequence alignment as a template.
(0.02 MB TXT)

Figure S5 Bootstrap support for all nodes was >80%. The phylogram represents a consensus tree and branch lengths are therefore not given. A maximum parsimony phylogram gave the same topology.
(0.09 MB TIF)

Figure S6 Bootstrap support for all nodes was >60%. The phylogram represents a consensus tree and branch lengths are therefore not given. A maximum parsimony phylogram gave the same topology.
(0.08 MB TIF)

Figure S7 (0.02 MB TXT)

Figure S8 (0.05 MB TXT)

Figure S9 (0.05 MB TXT)

Figure S10 Codons are labeled as “# site”. Mutations C50R, N51I, C59R, S108N and I164L correspond to codons 45, 46, 54, 103 and 159, respectively.
(0.06 MB TXT)
