# Supplementary material for: Evolutionary Patterning: A Novel Approach to the Identification of Potential Drug Target Sites in Plasmodium falciparum
Source: PLoS One. 2008 Nov 10;3(11):e3685. doi: 10.1371/journal.pone.0003685 (PMC2577034; doi:10.1371/journal.pone.0003685)
Supplement: Figure S1 — Sequences were retrieved from three databases: NCBI (http://www.ncbi.nlm.nih.gov), OrthoMCL (http://www.orthomcl.org/cgi-bin/OrthoMclWeb.cgi), and PlasmoDB version 5.3 (http://plasmodb.org). (0.05 MB DOC) [file pone.0003685.s001.doc]

| **BACTERIA** | | |
| --- | --- | --- |
| ORGANISM | ACCESSION NUMBER | DATABASE |
| *E. coli* | NP_418361 | NCBI |
| *B. subtilis* | Y14079 | NCBI |
| *S. aureus* | NP_371825.1 | NCBI |
| *S. pneumo* | NP_346597.1 | NCBI |
| *C. perfringens* | NP_563468.1 | NCBI |
| *T. maritima* | NP_229230.1 | NCBI |
| **PROTOZOA** | | |
| ORGANISM | ACCESSION NUMBER | DATABASE |
| *T. annulata* | XM_946898 | NCBI |
| *T. parva* | the|526.m04170 | OrthoMCL |
| *P. falciparum* | PF13_0269 | PlasmoDB |
| *P. chabaudi* | PC001056.02.0 | PlasmoDB |
| *P. yoelii* | PY00935 | PlasmoDB |
| *P. vivax* | Pv083470 | PlasmoDB |
| *P. berghei* | PB001520.02.0 | PlasmoDB |
| *P. knowlesi* | PKH_120240-1 | PlasmoDB |
| *T. brucei* | AF132295 | OrthoMCL |
| *L. major* | Lmj|F35.3080 | OrthoMCL |
| *E. histolytica* | ehi|34.m00232 | OrthoMCL |
| *G. lamblia* | gla|8173 | OrthoMCL |
| **METAZOA** | | |
| ORGANISM | ACCESSION NUMBER | DATABASE |
| *H. sapiens* | NM_203391 | NCBI |
| *M. musculus* | NM_008194 | NCBI |
| *R. norvegicus* | NM_024381 | NCBI |
| *M. mulatta* | XM_001100778 | NCBI |
| *D. rerio* | NM_001114584 | NCBI |
| *B. mori* | NP_001108335 | NCBI |
| *A. gambiae* | CAD27929.1 | NCBI |
| *D. melanogaster* | AY051456 | NCBI |
| *A. mellifera* | XP_623440 | NCBI |
| *B. taurus* | AAI22693 | NCBI |
