# Supplementary figures and images for: Evolutionary Patterning: A Novel Approach to the Identification of Potential Drug Target Sites in Plasmodium falciparum
Source: PLoS One. 2008 Nov 10;3(11):e3685. doi: 10.1371/journal.pone.0003685 (PMC2577034; doi:10.1371/journal.pone.0003685)

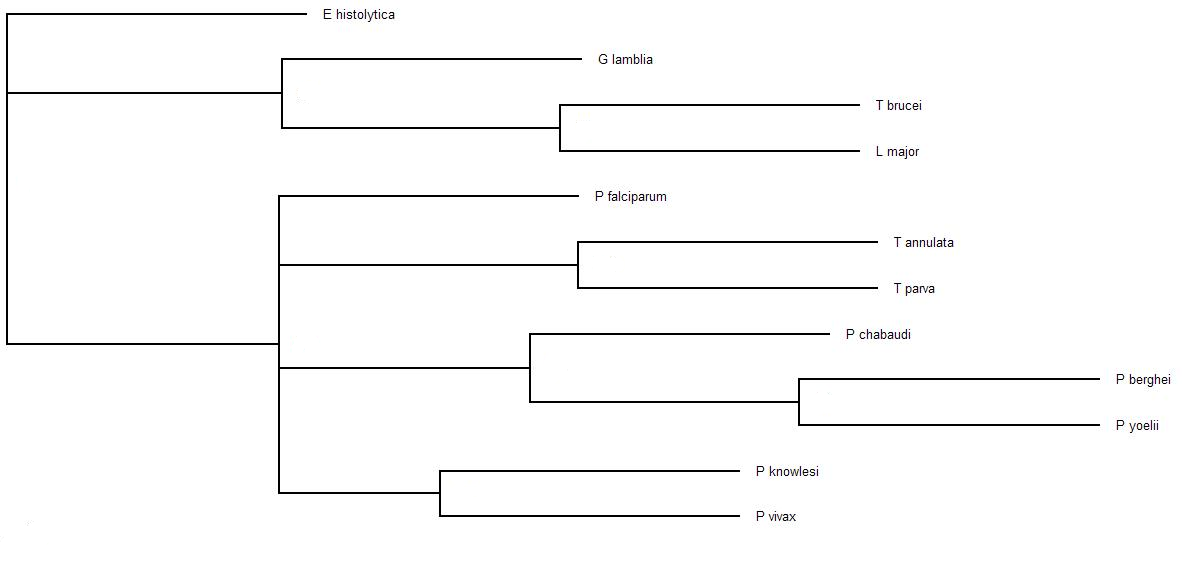

Supplement: Figure S5 — Bootstrap support for all nodes was >80%. The phylogram represents a consensus tree and branch lengths are therefore not given. A maximum parsimony phylogram gave the same topology. (0.09 MB TIF) [file pone.0003685.s005.tif]

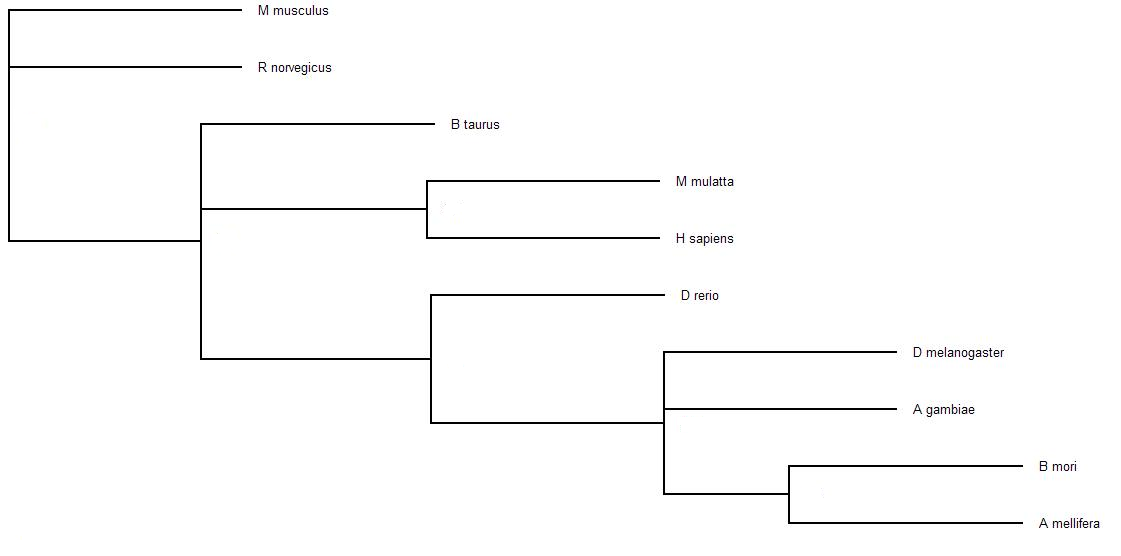

Supplement: Figure S6 — Bootstrap support for all nodes was >60%. The phylogram represents a consensus tree and branch lengths are therefore not given. A maximum parsimony phylogram gave the same topology. (0.08 MB TIF) [file pone.0003685.s006.tif]
